# OJIP-Derived Indices of Photochemical Characteristics in Sunflower and Maize: A Crop Type- and Cultivar-Level Assessment

**DOI:** 10.3390/plants15213379

**Published:** 2026-11-01

**Authors:** Guangrong Cai, Yanchuan Gong, Xiaotong Zeng, Feifei Li, Jie Liu, Jiayu You

**Affiliations:** 1 School of Chemistry and Chemical Engineering, Sichuan University of Arts and Science, Dazhou 635000, China; 2 Key Laboratory of Exploitation and Study of Distinctive Plants in Education Department of Sichuan Province, Dazhou 635000, China; 3 Henan Acdemy of Sciences, Zhenzhou 450002, China

**Keywords:** OJIP transient, maize, photosystem, sunflower

## Abstract

All oxygenic photosynthetic organisms rely on photosystems (PS) for light-dependent reactions. While C4 crops typically demonstrate greater photosynthetic capacity compared to C3 crops, differences in PS functions between these two types remain poorly resolved. Here, we used fast chlorophyll a fluorescence induction (OJIP transient) to derive indices of PS functions in five cultivars each of C3 sunflower and C4 maize, comparing performance at both species and cultivar levels. OJIP transients provide robust phenotypic indices of PSII performance (photosynthetic performance index, PIabs) and relative PSI electron transfer efficiency (estimated as the probability of electron transfer from Q_B_ to PSI acceptors, ΔV_IP_/ΔV_JP_). Despite lower relative chlorophyll reading (SPAD), sunflowers exhibited higher PIabs, averaging 2.20 versus 1.16 in maize. PIabs also varied significantly among cultivars within each species (1.00–1.38 in maize; 1.85–2.64 in sunflower), yet this intraspecific variation was considerably smaller than the twofold difference between crop types. The ΔV_IP_/ΔV_JP_ ratio was 25.65% higher in sunflower than in maize, and although cultivar-level variation was significant, the species contrast remained dominant. SPAD differed significantly among maize cultivars (54.8–65.4) but not among sunflower cultivars, with maize consistently higher (62.3 vs. 42.3). Principal component analyses further showed that SPAD was positively related to PIabs and negatively related to ΔV_IP_/ΔV_JP_ in both crops. Together, our findings reveal pronounced differences in photochemical characteristics between sunflower and maize, with differences between crop types larger than cultivar-level variation.

## 1. Introduction

Higher plants possess two photosystems (PS), namely PSI and PSII, which are embedded in thylakoid membranes. These photosystems drive electron transport [1] and generate biochemical reductant (NADPH_2_) and chemical energy (ATP), both essential for carbon assimilation [2]. It is widely accepted that C4 plants have superior photosynthetic efficiency compared to C3 plants [3,4], primarily due to the suppression of their photorespiratory pathway [5] and the presence of a CO_2_ concentration mechanism [6]. However, despite these known benefits, comparative assessments of the photochemical characteristics in C3 and C4 crops using robust phenotypic indices remain relatively limited.

Chlorophyll a fluorescence induction (OJIP transient) provides a rapid and comprehensive characterization of leaf photosynthetic performance by offering information on light energy absorption, excitation energy trapping, electron transport between photosystem II (PSII) and photosystem I (PSI), and the reduction in PSI end electron acceptors [7]. The photosynthetic performance index (PIabs), derived from the JIP test, integrates information on light energy absorption, excitation energy trapping, and conversion to electron transport, thereby reflecting the overall activity of PSII [8]. In addition, ΔV_IP_, defined as the amplitude of the relative variable fluorescence of the I-to-P rise, serves as a semi-quantitative indicator of PSI content [9], while the ratio ΔV_IP_/ΔV_JP_ is an index to reflect the efficiency with which an electron can move from the reduced intersystem electron acceptors to the PSI end electron acceptors [10]. Importantly, the OJIP measurement is performed on dark-adapted samples and completed within one second [11,12], making it a highly efficient tool for comparative studies of PS functions.

Yet a survey of the literature reveals considerable inconsistencies in reported PIabs values. For C3 rice, PIabs has ranged from 2 to 4 across diverse germplasm [13], whereas values between 1.5 and 3 have been reported in another study [14]. In cotton, a maximum PIabs of 7.55 has been recorded [15], while cucumber shows substantially lower values of approximately 1.0 [16]. For C4 maize, PIabs has been reported to range from 1.5 to 4 [17], though values between 1 and 3 have also been documented [18]. Critically, however, these estimates are derived from independent studies. The only direct comparison available is that of Killi, et al. [19], who measured PIabs in sunflower and maize using two cultivars per crop. However, their design centered on drought and heat stress responses, so it does not provide a common-condition, multi-cultivar comparison of basal PS activity between the two crop types.

Maize (*Zea mays* L.) is a globally important staple crop with a C4 photosynthetic pathway that enables high productivity in warm climates. Sunflower (*Helianthus annuus* L.) is a widely grown oilseed crop valued for its adaptability and high-quality oil production. In this work, five representative cultivars of each species were cultivated, allowing photochemical characteristics to be examined at two levels: between crop types (sunflower vs. maize) and among cultivars within each species. The study aims to: (1) investigate the OJIP transient to identify crop-specific OJIP curves; (2) compare the photosystem (PS) functions between crops and cultivars; and (3) assess whether observed differences are linked to chlorophyll content. In NADP-ME-type C4 maize, the fixation of one CO_2_ requires 5 ATP and 2 NADPH, whereas C3 sunflower requires only 3 ATP and 2 NADPH [20]. This stoichiometric difference implies a substantially higher ATP demand per CO_2_ fixed in maize. We therefore hypothesized that maize would exhibit higher OJIP-derived indices than sunflower, reflecting a greater need for electron transport and ATP synthesis to support its photosynthetic metabolism.

## 2. Results

### 2.1. OJIP Curves and JIP-Test Parameters

**Figure 1:**
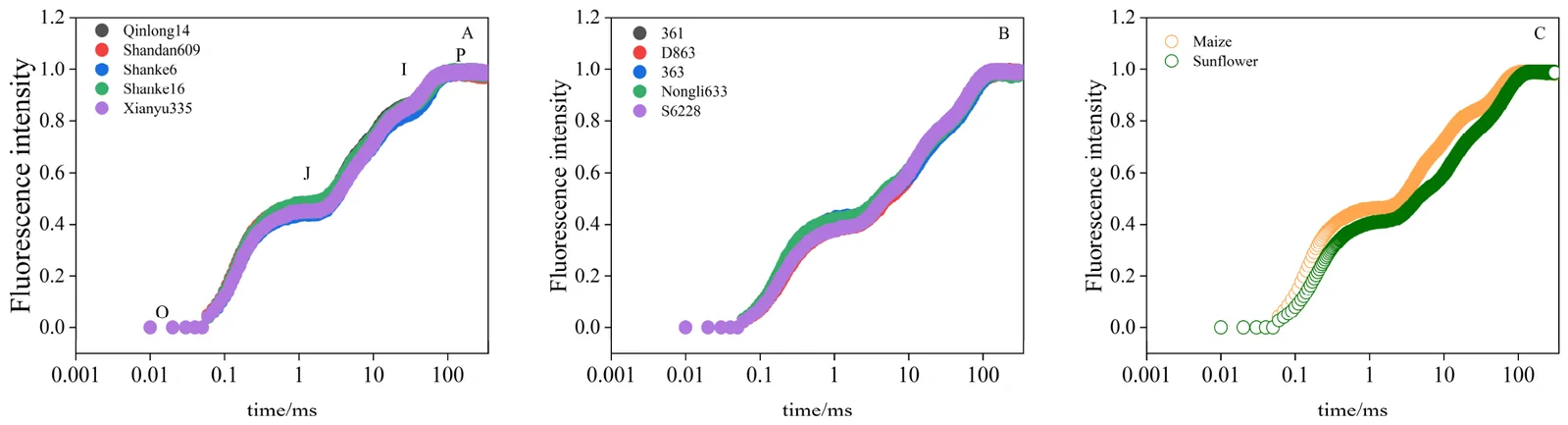
**Normalized OJIP fluorescence induction curve**. (**A**) Averaged OJIP curves of five maize cultivars; (**B**) averaged OJIP curves of five sunflower cultivars; (**C**) comparison of averaged OJIP curves between sunflower and maize.

**Figure 2:**
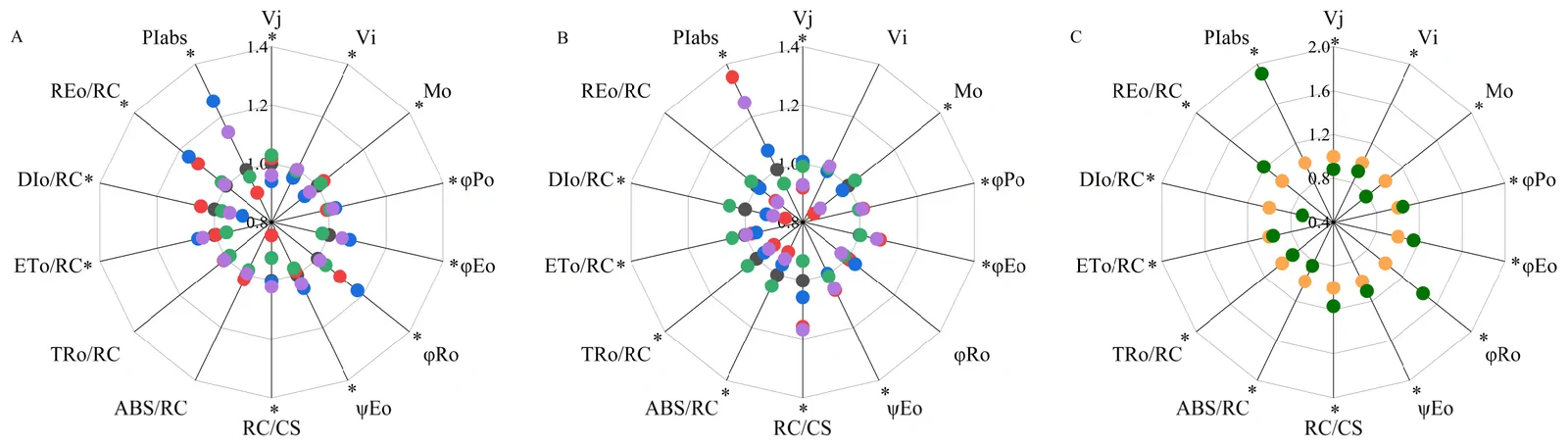
**Spider plots of JIP-test parameters.** (**A**) Averaged parameter values of five maize cultivars; (**B**) Averaged parameter values of five sunflower cultivars; (**C**) Comparison of averaged parameter values between sunflower and maize. For each parameter in panels A and B, the value of a single reference cultivar (black circle; Qinlong 14 for maize, 361 for sunflower) is set to 1, and all other values are expressed relative to this reference. For each parameter in panel (**C**), the values for maize (Orange-yellow) are set to 1, and the values for sunflower (Green) are expressed relative to this reference. Asterisks (*) denote statistically significant differences between cultivars or crop types at the 0.05 probability level.

As depicted in Figure 1, the leaves of sunflower and maize exhibited polyphasic patterns in chlorophyll a fluorescence. Notably, the ‘J’ and ‘I’ steps in sunflower were markedly lower compared to maize (Figure 1C), indicating species-specific differences in the redox behavior of the intersystem electron transport chain. Figure 2A illustrates the relative values of the JIP test parameters for the five maize cultivars, revealing significant differences (*p* < 0.05) in Vj, Vi, Ψ_Eo_, Mo, φPo, φEo, φRo, ETo/RC, REo/RC, DIo/RC, PIabs and RC/CS among cultivars. Although φPo is widely used as an indicator of photoinhibition, its values varied only narrowly, from 0.79 (Qinlong 14) to 0.81 (Shanke 6) (Table S1), suggesting that none of the cultivars suffered substantial photodamage under the experimental conditions. Ψ_Eo_ ranged from 0.53 (Shanke 16) to 0.57 (Shanke 6). PIabs showed a wider spread, from 1.00 (Shandan 609) to 1.38 (Shanke 6) (Table S1), indicating significant differences in PSII activity among the cultivars.

For sunflowers, significant differences were found among cultivars in Vj, Ψ_Eo_, Mo, φPo, φEo, ETo/RC, DIo/RC, PIabs, RC/CS, ABS/RC and TRo/RC (Figure 2B). Specifically, φPo ranged from 0.826 (Nongli 633) to 0.839 (D863), and Ψ_Eo_ ranged from 0.58 (363) to 0.62 (D863) (Table S2). ABS/RC varied from 3.26 (D863) to 3.72 (Nongli 633), while PIabs spanned a wider range, from 1.85 (Nongli 633) to 2.64 (D863) (Table S2). When the two crops were compared, significant differences (*p* < 0.05) were observed across all JIP test parameters (Figure 2C). The most pronounced difference occurred in PIabs: sunflower reached 2.20, which is 1.9 times higher than the value of 1.16 recorded in maize.

**Figure 3:**
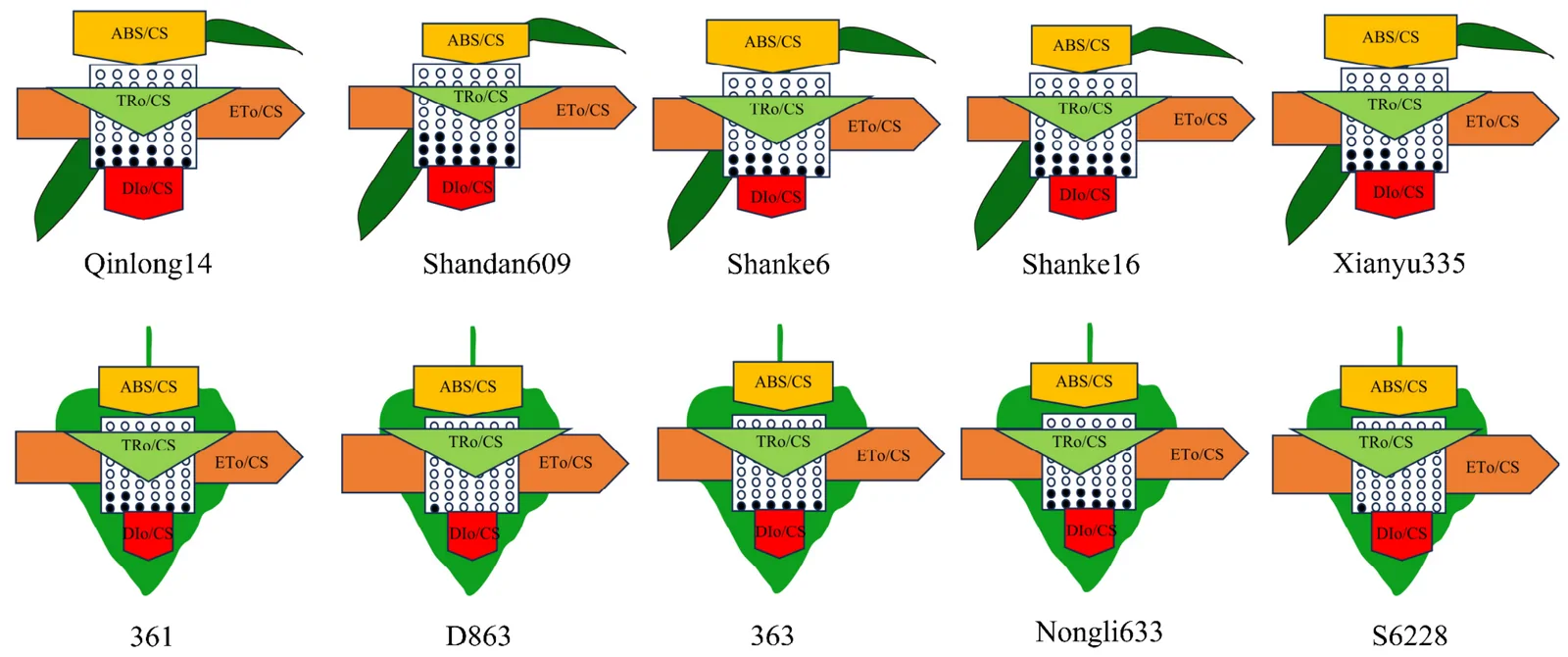
Energy pipeline leaf model of phenomenological fluxes (per cross-section, CS) in maize and sunflower. Each value of the measured parameters is the mean, and the width of each arrow corresponds to the relative intensity of the flux. ABS/CS, absorption flux per CS; TRo/CS, trapped energy flux per CS; ETo/CS, electron transport flux per CS; DIo/CS, dissipated energy flux per CS; circles inscribed in squares—RC/CS, % of active/inactive reaction centers.

To visualize OJIP-based energy flux, such as electron transport flux per cross section (ETo/CS), dissipated energy flux per cross section (DIo/CS), density of reaction centers (RC/CS), absorption flux per cross section (ABS/CS), and trapped energy flux per cross section (TRo/CS), energy pipeline models were developed for sunflower and maize (Figure 3). Within maize, ETo/CS ranged from 0.44 (Shandan 609) to 0.56 (Shanke 6), whereas within sunflower it ranged from 0.53 (Nongli 633) to 0.63 (S6228). Similarly, DIo/CS varied from 0.23 (Shanke 16) to 0.26 (Qinlong 14) in maize and from 0.19 (363) to 0.20 (S6228) in sunflower. RC/CS ranged from 0.25 (Shandan 609) to 0.30 (Xianyu 335) in maize and from 0.29 (Nongli 633) to 0.36 (S6228) in sunflower. On average, maize showed lower ETo/CS and RC/CS (0.51 and 0.28, respectively) but higher DIo/CS (0.24) than sunflower (0.57, 0.33, and 0.19, respectively). Thus, relative to maize, sunflower exhibited a higher electron transport flux and a greater density of reaction centers, accompanied by a lower dissipated energy flux per cross section.

**Figure 4:**
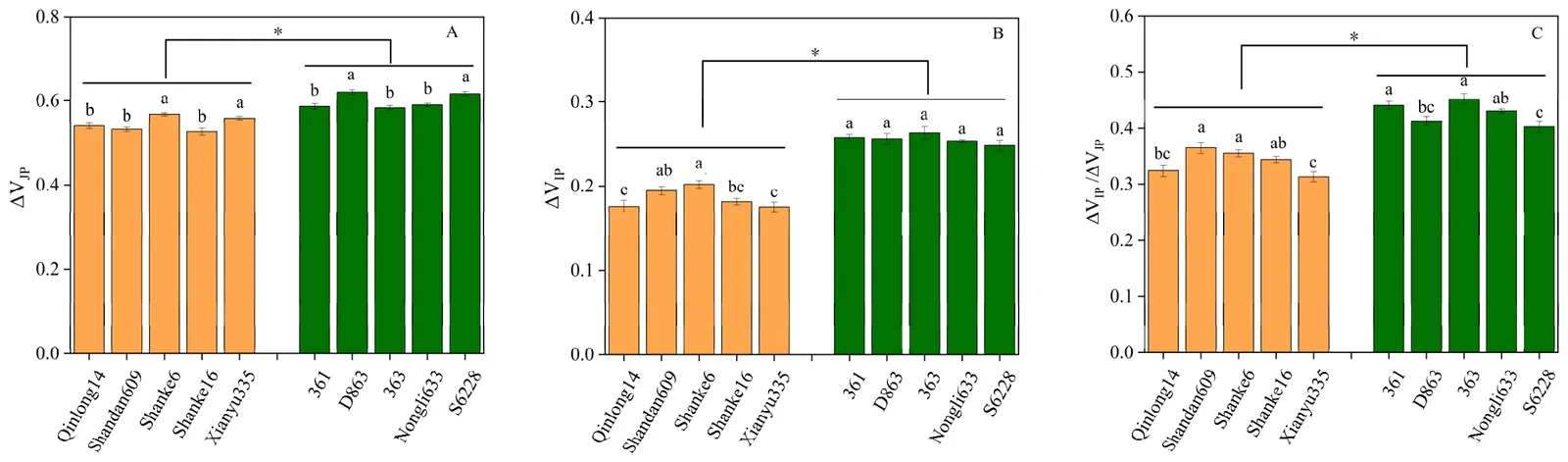
**Comparison of JP amplitude, IP amplitude and ΔV_IP_/ΔV_JP_ in maize and sunflower.** (**A**) Averaged JP amplitude; (**B**) averaged IP amplitude; (**C**) ΔV_IP_/ΔV_JP_. Asterisks (*) denote statistically significant differences between maize and sunflower at the 0.05 probability level. Different letters indicate significant differences among cultivars at the 0.05 level. Orange-yellow denotes maize, whereas green denotes sunflower. Data are presented as means ± SE (*n* = 3).

Comparison of ΔV_JP_ revealed significant differences among both maize and sunflower cultivars (Figure 4A). Interestingly, significant differences in ΔV_IP_ among cultivars were detected only in maize (Figure 4B). The mean ΔV_IP_/ΔV_JP_ ratio was 0.34 for maize and 0.42 for sunflower, and a t-test indicated a significant difference between the two crops (Figure 4C).

### 2.2. Chlorophyll Content in Maize and Sunflower

**Figure 5:**
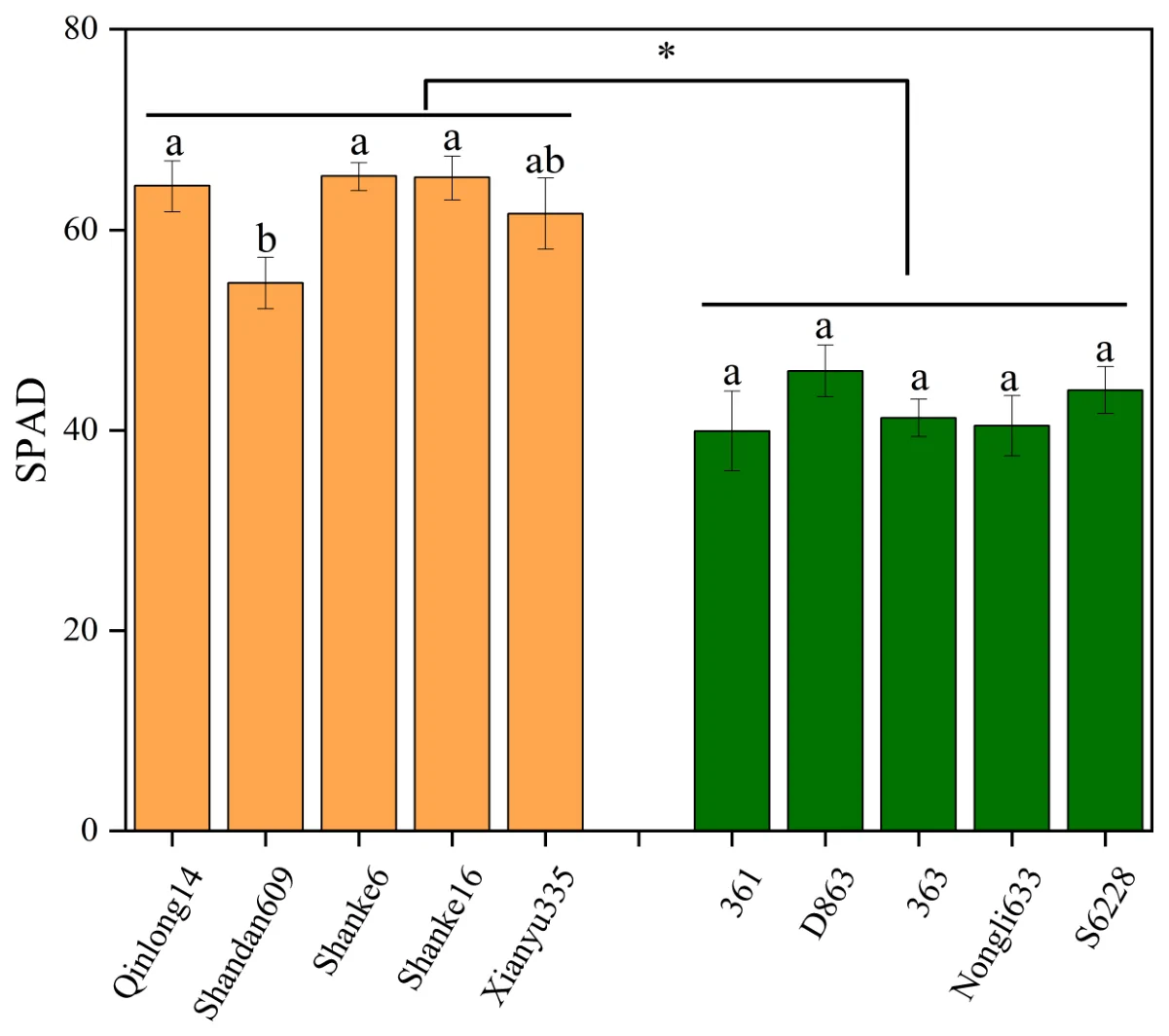
**Relative chlorophyll reading in maize and sunflower.** Asterisks (*) denote statistically significant differences between maize and sunflower at the 0.05 probability level. Different letters indicate significant differences among cultivars at the 0.05 level. Orange-yellow denotes maize, whereas green denotes sunflower. Data are presented as means ± SE (*n* = 3).

Relative chlorophyll reading, measured by SPAD, showed a significant difference among maize cultivars (*p* < 0.05). The highest SPAD value was observed in Shanke16 (65.4), while the lowest was in Shandan609 (54.8). For sunflower, no significant differences were found in SPAD values. Overall, maize exhibited a higher average SPAD than sunflower (*p* < 0.05), with averages of 62.3 and 42.3, respectively (Figure 5).

### 2.3. Linkages Between Leaf Relative Chlorophyll Reading and PSII Activity

**Figure 6:**
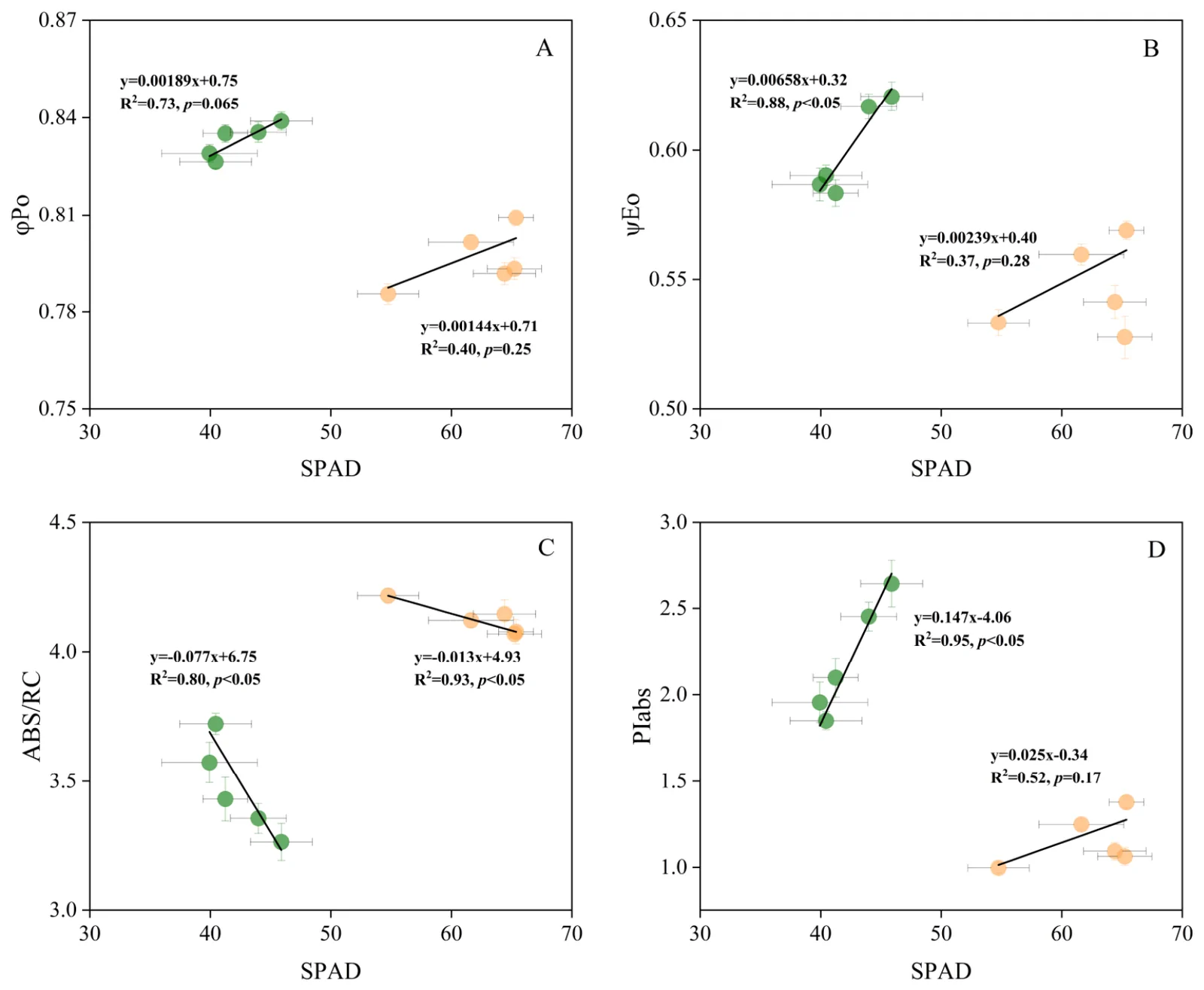
**Relationship between SPAD values and PSII activity parameters in maize and sunflower.** (**A**) Maximum quantum yield of primary photochemistry (φPo). (**B**) Quantum yield of electron transport beyond Q_A^−^_ (ψEo). (**C**) Light absorption flux per active reaction center (ABS/RC). (**D**) Performance index on an absorption basis (PIabs). Each panel shows the correlation between SPAD values and the corresponding PSII parameter across all measured cultivars of both species. Orange-yellow denotes maize, whereas green denotes sunflower. Each point represents the mean value of photochemical traits (*n* = 5 per species).

Since φPo, ψEo, and ABS/RC are the components underlying PIabs, we examined how SPAD relates to each of these parameters as well as to PIabs itself, using linear regression analysis (Figure 6). SPAD was positively related to the probability that a trapped exciton moves an electron into the electron transport chain beyond Q_A^−^_ (ψEo) in sunflower (y = 0.00658x + 0.32; R^2^ = 0.89, *p* < 0.05), whereas no significant relationship was observed in maize (*p* = 0.31) (Figure 6B). A significant negative relationship was found between SPAD and ABS/RC in both sunflower (y = −0.077x + 6.75; R^2^ = 0.80, *p* < 0.05) and maize (y = −0.013x + 4.93; R^2^ = 0.93, *p* < 0.05) (Figure 6C). In addition, SPAD was positively related to PIabs in sunflower (y = 0.147x − 4.06; R^2^ = 0.95, *p* < 0.05). These results indicated that, while SPAD was consistently linked to ABS/RC in both species, its association with ψEo and PIabs was species-dependent, being significant only in sunflower.

### 2.4. Principal Component Analysis (PCA) Analysis

**Figure 7:**
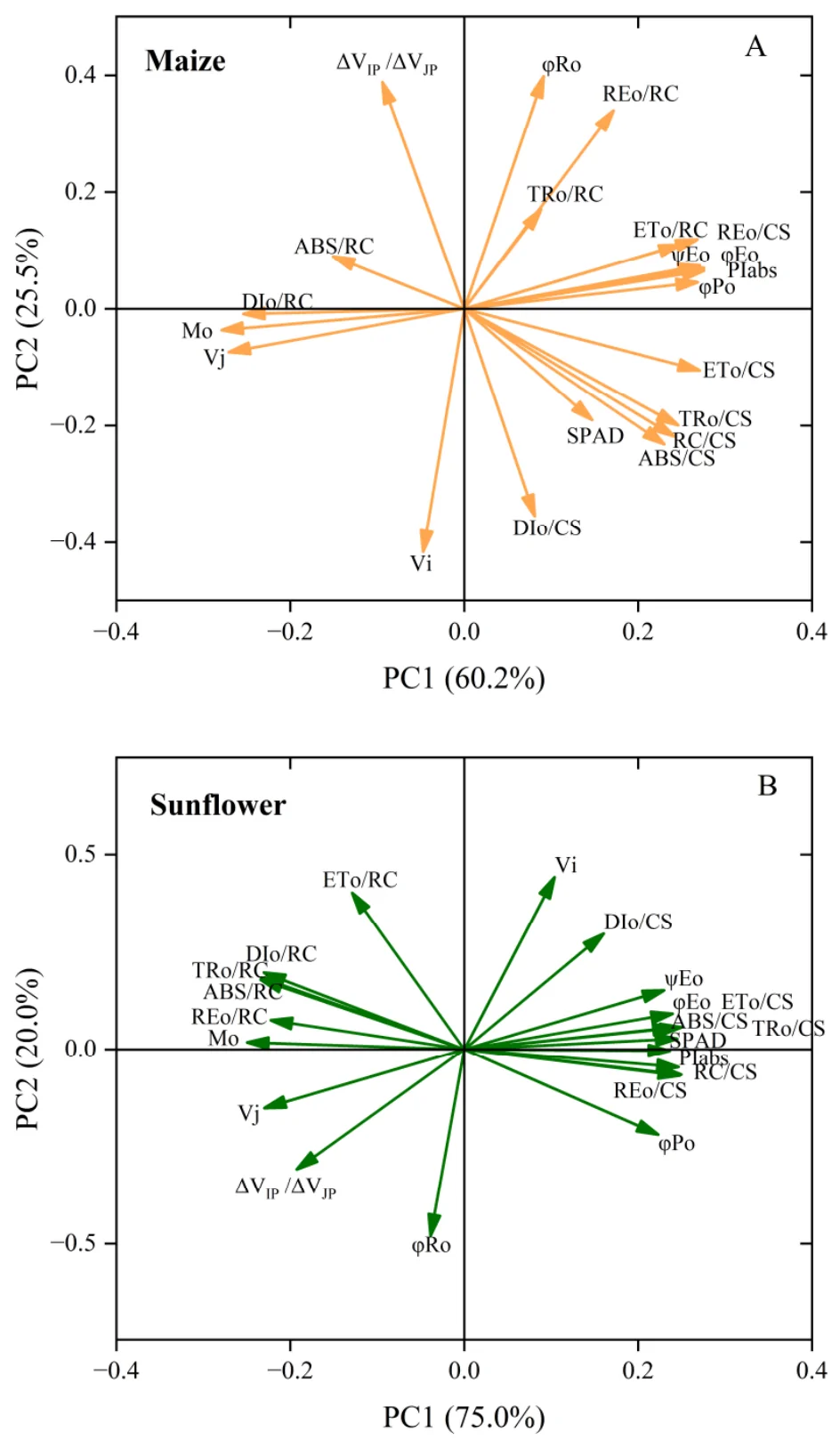
Loading plots of principal component analysis (PCA) on various attributes in maize (**A**) and sunflower (**B**).

Furthermore, PCA was applied to quantify the correlations between SPAD and PS functions (Figure 7). In the loading plot, a sharp angle indicates a positive correlation between parameters, while an obtuse angle represents a negative correlation. For maize, the first principal component (PC1) explained 60.2% of the total variance, while the second principal component (PC2) explained 25.5% of the variance. For sunflower, the first principal component (PC1) explained 75.0% of the total variance, while the second principal component (PC2) explained 20.0% of the variance. SPAD and PIabs were positively related in both crops, with a stronger correlation observed in sunflower, as indicated by the smaller angle between these parameters. This supported the results from the linear regression analysis. In contrast, SPAD was negatively related to ΔV_IP_/ΔV_JP_ in both crops.

## 3. Discussion

**Figure 8:**
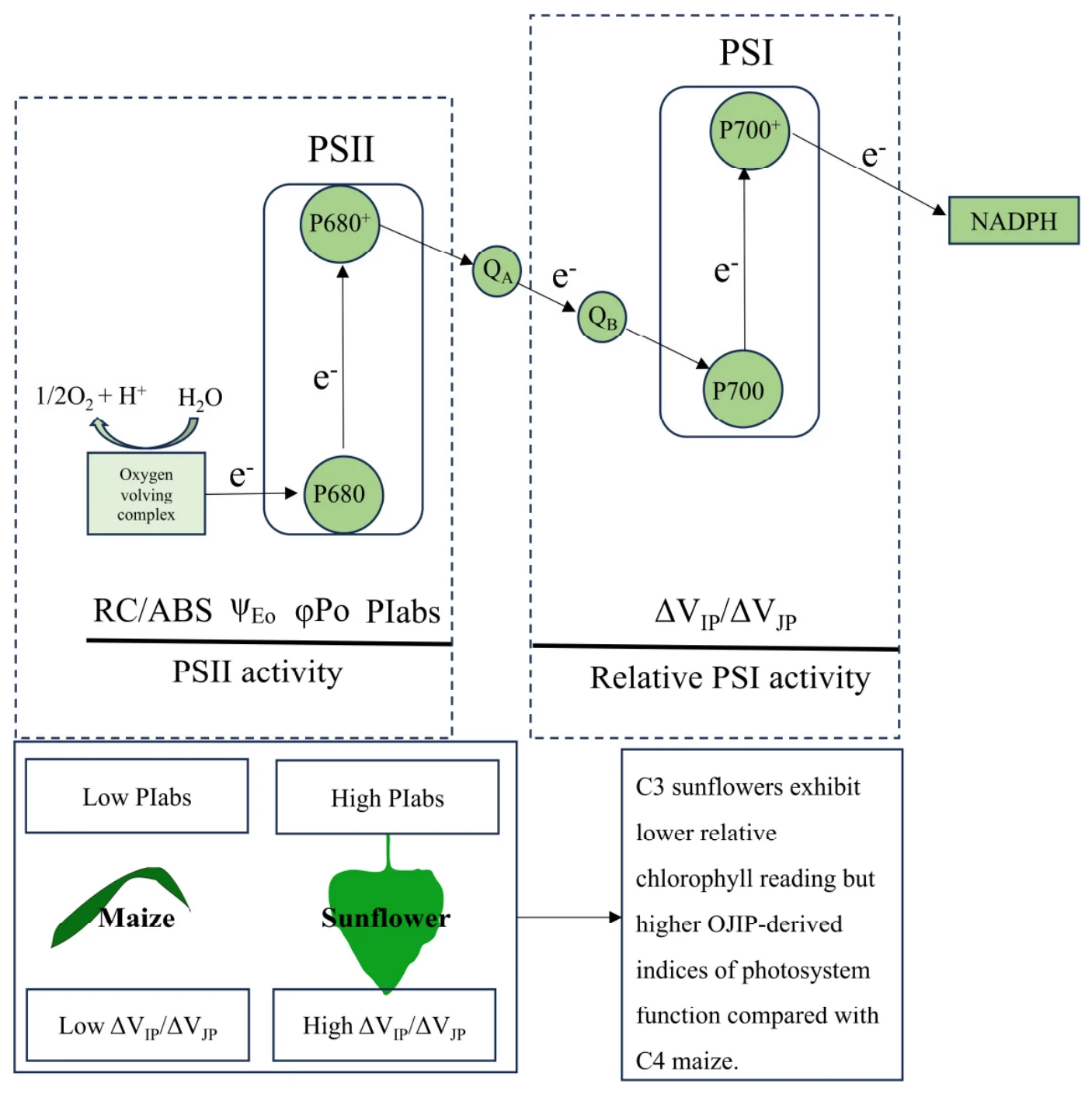
A conceptual diagram explains the differences in photochemical properties between sunflowers and maize. Linear electron transport proceeds from H_2_O to NADP+ through four major thylakoid membrane protein complexes: (i) photosystem II (PSII), including the oxygen-evolving complex (OEC), where water is oxidized to O_2_, releasing electrons and protons; (ii) the plastoquinone (PQ) pool, which carries electrons from PSII to the cytochrome b6f complex; (iii) the cytochrome b6f complex (Cyt b6f), which transfers electrons to plastocyanin (PC); and (iv) photosystem I (PSI), where electrons are re-energized and transferred via ferredoxin (Fd) to ferredoxin-NADP^+^ reductase (FNR), which reduces NADP^+^ to NADPH.

By combining fast chlorophyll a fluorescence induction with the JIP test, we compared the photochemical characteristics of sunflower and maize at two levels: between the two crops and among the five cultivars within each crop. Contrary to our hypothesis, we found higher PS functions observed in sunflowers based on OJIP-derived indices (Figure 8). Significant differences among cultivars were also observed, but differences between crop types were greater than cultivar-level variation.

### 3.1. Higher PSII Activity in C3 Sunflower

PIabs is a multi-parameter metric that accounts for different photosynthetic steps. Thus, decreases in the efficiency of any given process can potentially impact PIabs. In the study, we observed higher values for φPo, RC/ABS and ψEo in sunflower compared to maize (Figure 2C). According to Björkman and Demmig [21], φPo is highly conserved within species. In our study, φPo was higher in sunflower (0.83) than in maize (0.80), indicating a difference between crop types in PSII photochemistry [22]. Hermans, et al. [23] described RC/ABS as the probability that a chlorophyll molecule is involved in the reaction centers. A previous study found RC/ABS was negatively related to Mo (the rate of Q_A_ reduction) [24], which helps explain the observed difference in RC/ABS between sunflower and maize (Figure 2C). Additionally, a reduced ‘J’ step in sunflower (Figure 1C) indicated a lower accumulation of Q_A^−^_, indicating improved electron transport efficiency [15]. Overall, the higher PIabs in C3 sunflower can be attributed to all three of its components.

Chlorophyll is a key biochemical component in photosynthesis, responsible for absorbing, transporting, and converting light energy [25]. C4 plants are generally considered to invest more nitrogen into light-harvesting proteins than C3 plants [25], which would predict a higher chlorophyll content and, presumably, a greater light-harvesting capacity in maize. Interestingly, however, our study found an apparent paradox: sunflower (C3) had a lower relative chlorophyll reading but a higher PIabs than maize (C4) (Figure 2C, Figure 5 and Figure 6D), indicating that PSII activity is not determined solely by pigment content. Consistent with this view, previous studies have shown that reducing chlorophyll content can prevent excessive light absorption, enhance PSII efficiency, and lead to a higher electron transport rate [26,27,28]. In other words, a higher chlorophyll content does not necessarily translate into higher photosynthetic efficiency. Notably, we also found that most reaction centers in sunflower were active, whereas the dissipated energy flux per cross section (DIo/CS) was higher in maize than in sunflower (Figure 3), suggesting that sunflower uses a greater fraction of absorbed light for photochemistry.

Interestingly, we observed a negative relationship between SPAD and ABS/RC in both crops (Figure 6C). Previously, weak negative relationships were reported in grapevine [29] and tomato [30], whereas a significant negative relationship was observed in rice [31].

### 3.2. Higher ΔV_IP_/ΔV_JP_ in Sunflower

In the study, we compared ΔV_IP_/ΔV_JP_ between crop types and cultivars; this parameter reflects the probability of an electron transfer from Q_B_ to PSI acceptors. Sunflower showed a higher ΔV_IP_/ΔV_JP_ than maize (Figure 4C). Notably, this does not necessarily indicate higher PSI activity. PSI activity is most directly assessed by evaluating cyclic electron flow (CEF) activity [32,33,34], in which the Փ_PSI_/Փ_PSII_ ratio serves as an indicator of CEF activation. Interestingly, previous work found that ΔV_IP_/ΔV_JP_ was negatively correlated with the Փ_PSI_/Փ_PSII_ ratio [35]. Their work also revealed a strong negative correlation between ΔV_IP_/ΔV_JP_ and the plant biomass in bread wheat and durum wheat. There is broad consensus that C4 plants possess a higher CEF capacity than C3 plants in order to meet the additional ATP requirements of C4 photosynthesis [20]. In this case, the higher ΔV_IP_/ΔV_JP_ in sunflower may be reasonably explained. However, further work is needed to verify this, which represents an important limitation of the present study.

### 3.3. Cultivar Difference vs. Crop Type Difference

Previous works have highlighted substantial variation in PS functions among crop cultivar [13,36], and our results are broadly consistent with this view: significant cultivar-level differences were detected in most JIP-test parameters for both maize and sunflower (Figure 2A,B). However, a closer comparison reveals that the magnitude of this intraspecific variation was considerably smaller than the divergence observed between the two crop species. For example, PIabs varied only from 1.00 to 1.38 among the five maize cultivars and from 1.85 to 2.64 among the sunflower cultivars, whereas the species means differed by nearly twofold. Thus, although cultivar selection clearly modulates PSII performance, crop type appears to exert a stronger influence on the photochemical traits measured here. This difference is likely rooted in the fundamental physiological divergence: maize and sunflower differ in their CO_2_-concentrating mechanisms, ATP/NADPH stoichiometry, and the balance between linear and cyclic electron flow [37,38]. It should be noted, however, that this comparison was conducted under only one growth condition; the extent to which cultivar and crop-type differences are expressed under stressful conditions remains unmeasured. Such differences may nonetheless become apparent under stress, as reported by Killi, et al. [39].

## 4. Materials and Methods

### 4.1. Experimental Site and Design

A field experiment was conducted at Dali in 2023. The experimental site has a warm temperate, semi-humid monsoon climate, with a mean annual temperature of 14.4 °C. The soil has an ECe of 2.3 dS/m and a pH of 8.0. Precipitation from May to August was 99.2, 176.2, 106, and 91.3 mm, respectively. No irrigation was applied during the measurement period. The experiment was arranged in a randomized complete block design with three replicates. Within each block, plots were randomly allocated to five maize cultivars (Qinlong14, Shandan609, Shanke16, Shanke6, and Xianyu335) and five sunflower cultivars (361, D863, 363, Nongli633, and S6228). In each replicate, each cultivar was grown in three rows, each 50 m long, with 50 cm inter-row spacing. Maize was planted at a density of approximately 90,000 seeds ha^−1^ and sunflower at 67,500 seeds ha^−1^. Chemical fertilizers were applied at 240 kg N ha^−1^ and 90 kg P_2_O_5_ ha^−1^. Measurements were conducted during the flowering stage in August.

### 4.2. OJIP Transient Measurement

The OJIP transient was measured using the Dual-PAM-100 system (Heinz Walz, Effeltrich, Germany), with fluorescence detected at a resolution of 10 μs. It should be noted that dedicated PEA (Plant Efficiency Analyzer) fluorometers (e.g., Hansatech, Photon Systems Instruments) are also commonly employed for high-resolution OJIP measurements. A 320 ms pulse of red light (650 nm, 2000 μmol photons m^−2^ s^−1^) induced the transient, and the fluorescence signal was recorded continuously over the pulse to capture the full O-J-I-P polyphasic rise. Measurements were performed on the upper surfaces of the youngest fully expanded leaves. Within each block, 2–3 plants per cultivar were selected for OJIP measurement, and a total of 8 plants per cultivar were assessed across the three blocks. These plants were treated as subsamples within each plot, and the plot was the experimental unit. For each measurement (01:00), the leaf was detached, and the measurement was completed rapidly within 3 min at ambient temperature (~23 °C). Measurement in natural darkness first ensured that the PSII reaction centers were in an “open” state [40], while minimizing water loss from the detached leaves.

The JIP-test analysis of OJIP curves yields a range of fluorescence parameters, which are categorized into basic fluorescence data, quantum efficiencies, specific energy fluxes per reaction center (RC), phenomenological energy fluxes per cross-section (CS), and performance indices (Table 1) [7,8]. Basic fluorescence parameters were recorded at Fo (20 μs), Fj (2 ms), Fi (30 ms), and Fm (maximal). The OJIP fluorescence transient was doubly normalized at Fo and Fm for visualization. The maximum quantum yield of PSII primary photochemistry, φPo, is expressed as φPo = 1 − (Fo/Fm). The probability that an electron progresses beyond Q_A^−^_ is defined as Ψ_Eo_ = ETo/TR = 1 − Vj. PSII activity was calculated as: PIabs = (RC/ABS) × [φPo/(1 − φPo)] × [ψEo/(1 − ψEo)]; where RC/ABS was density of reaction centers. PSI activity was also estimated, shown as the ratio of relative fluorescence at steps J (ΔV_JP_ = 1 − Vj) and I (ΔV_IP_ = 1 − Vi) [35].

### 4.3. Chlorophyll Content Measurement

The relative chlorophyll reading (SPAD) was determined using the MultispeQ (Michigan State University, East Lansing, MI, USA) with the “SPAD only” protocol, which quantifies the relative transmission of red (650 nm) and infrared (940 nm) light.

### 4.4. Statistical Analysis

Before analysis, the 2–3 plants sampled for OJIP determination within each plot were averaged to obtain a plot mean so that the three blocks served as independent replicates (*n* = 3 per cultivar). Cultivar (and species, where applicable) effects were tested using a linear model that included block and cultivar (species) as fixed effects, with the block × cultivar interaction as the error term. When species were compared, the cultivar was nested within the species.

Statistical analysis was performed using ANOVA followed by the t-test or Duncan’s multiple range test (*p*  <  0.05), using the SPSS 23.0 software (SPSS Inc., Chicago, IL, USA). Principal component analysis (PCA) was conducted using Origin 2024 software (Northampton, MA, USA). The analysis was based on the correlation matrix, which standardizes each variable to unit variance before extracting the principal components. Both regressions and PCA were performed on cultivar means. Data presented in graphs were shown as mean value  ±  standard error (SE).

## 5. Conclusions

The study compared the photochemical characteristics of sunflower and maize, both between the two species and among five cultivars within each crop, using JIP-test parameters derived from OJIP transients. Sunflower showed higher values of several JIP-test parameters than maize. The higher PSII activity in sunflower was attributed to its tendency to use a larger fraction of absorbed energy for photochemistry. The higher ΔV_IP_/ΔV_JP_ in sunflower indicates a greater relative amplitude of the IP phase; however, because Փ_PSI_/Փ_PSII_ was not directly determined in this study, this result cannot be attributed to lower PSI activity. Cultivar-level differences were detected in most JIP-test parameters, but their magnitude was smaller than the differences observed between the two species under the conditions tested.

Several limitations of this study should be acknowledged. First, the measurements were made under a single set of growth conditions and at a single developmental stage, which may have constrained the expression of cultivar-level variation. Second, our comparison was restricted to one C3 and one C4 crop species, so whether the observed patterns reflect a general C3-C4 dichotomy or are specific to the species examined remains an open question. Resolving this will require broadening the analysis to a wider range of C3 and C4 species. Third, only five cultivars were included in the regression analysis; future work should therefore incorporate a larger number of cultivars. Addressing these gaps will be essential for a more complete mechanistic understanding of the differences in photosynthetic performance between C3 and C4 crops.

## Figures and Tables

**Table 1:** JIP-test fluorescence parameters.

Fluorescence Parameters	Description
Fo = F_20μs_	Minimum fluorescence intensity at 20 μs
F_P_	Maximal fluorescence intensity
Fj = F_2ms_	Fluorescence intensity at the J step (2 ms)
Fi = F_30ms_	Fluorescence intensity at the I step (30 ms)
Vj = (Fj − Fo)/(Fm − Fo)	Relative variable fluorescence at 2 ms (J-step)
Vi = (Fi − Fo)/(Fm − Fo)	Relative variable fluorescence at 30 ms (I-step)
Ψ_Eo_ = ETo/TR = 1 − Vj = ΔV_JP_	Probability that a trapped exciton moves an electron into the electron transport chain beyond Q_A^−^_
Mo = 4(F_300μs_ − Fo)/(Fm − Fo)	The initial slope of the transient
ΔV_IP_ = 1 − Vi	Relative amplitude of the I-P phase
ΔV_IP_/ΔV_JP_ = (1 − Vi)/(1 − Vj)	Probabilities with an electron from Q_B_ is transferred until PSI acceptors (indicator of PSI activity)
φPo = [1 − (Fo/Fm)] = Fv/Fm	The maximum quantum yield for primary photochemistry
φEo = [1 − (Fo/Fm)] × (1 − Vj)	Quantum yield for electron transport
φRo = [1 − (Fo/Fm)] × (1 − Vi)	Quantum yield for reduction in the PSI end electron acceptors: Ferredoxin and NADP
ABS/RC = Mo × (1/V_j_) × (1/φPo)	Ratio of antenna chlorophyll to PSII reaction center chlorophyll (indicator of antenna size for PSII)
TRo/RC = Mo × (1/Vj)	Trapped energy flux per reaction center
ETo/RC = Mo × (1/Vj) × Ψ_Eo_	Electron transport flux per reaction center
REo/RC = φRo × ABS/RC	Reduction per reaction center: PSI end electron acceptors
DIo/RC = (ABS/RC) − (TRo/RC)	Dissipated energy flux per reaction center
RC/CS = φPo × (V_j_/Mo) × (ABS/CS)	Density of reaction centers
ABS/CS ≈ Fm	Absorption flux per excited cross-section (CS)
TRo/CS = φPo × (ABS/CS)	Trapped energy flux per CS
ETo/CS = φEo × (ABS/CS)	Electron transport flux per CS
REo/CS = φRo × (ABS/CS)	Reduction per CS: PSI end electron acceptors
DIo/CS = (ABS/CS) − (TRo/CS)	Dissipated energy flux per CS
PIabs = (RC/ABS) × [φPo/(1 − φPo)] × [ψ_Eo_/(1 − ψEo)]	Performance index on absorption basis (indicator of PSII activity)

## Data Availability

The data presented in this study are available on request from the corresponding author.

## Supplementary Materials

The following supporting information can be downloaded at: https://www.mdpi.com/article/10.3390/plants15213379/s1. Table S1. JIP-test parameters in five maize cultivars. Table S2. JIP-test parameters in five sunflower cultivars.
