# Supplementary material for: OJIP-Derived Indices of Photochemical Characteristics in Sunflower and Maize: A Crop Type- and Cultivar-Level Assessment
Source: Plants (Basel). 2026 Nov 1;15(21):3379. doi: 10.3390/plants15213379 (PMC13765843; doi:10.3390/plants15213379)
Supplement: Supplementary file 1 [file plants-15-03379-s001.zip › plants-4596480-supplementary.pdf]

Table S1. JIP-test parameters in five maize cultivars

	Qinlong 14	Shandan 609	Shanke 6	Shanke 16	Xianyu 335
Vj	0.459±0.006	0.467±0.005	0.431±0.004	0.473±0.008	0.440±0.004
Vi	0.824±0.007	0.806±0.005	0.798±0.005	0.819±0.004	0.825±0.006
Mo	1.506±0.036	1.545±0.012	1.421±0.014	1.525±0.029	1.455±0.021
φPo	0.792±0.003	0.786±0.003	0.809±0.002	0.793±0.003	0.802±0.002
φEo	0.429±0.006	0.419±0.005	0.460±0.003	0.419±0.007	0.449±0.003
φRo	0.139±0.006	0.153±0.004	0.164±0.003	0.144±0.003	0.141±0.005
Ψ_{Eo}	0.541±0.006	0.533±0.005	0.569±0.004	0.528±0.008	0.560±0.004
RC/CS	0.297±0.010	0.251±0.009	0.298±0.009	0.274±0.008	0.303±0.002
ABS/RC	4.145±0.055	4.216±0.027	4.077±0.047	4.069±0.029	4.121±0.027
TRo/RC	3.282±0.041	3.311±0.023	3.298±0.030	3.227±0.015	3.303±0.023
ETo/RC	1.775±0.018	1.766±0.026	1.876±0.024	1.702±0.026	1.848±0.011
DIo/RC	0.863±0.021	0.904±0.016	0.779±0.018	0.842±0.018	0.818±0.011
REo/RC	0.575±0.018	0.644±0.017	0.667±0.018	0.586±0.012	0.578±0.017
PIabs	1.093±0.051	0.997±0.030	1.377±0.034	1.063±0.051	1.248±0.030

Notes: These are the absolute values corresponding to the relative values shown in Figure 2A. Data are presented as means ± SE (n = 3).

Table S2. JIP-test parameters in five sunflower cultivars

	361	D863	363	Nongli633	S6228
Vj	0.413±0.006	0.379±0.006	0.417±0.005	0.41±0.004	0.383±0.005
Vi	0.742±0.004	0.743±0.006	0.736±0.007	0.746±0.001	0.752±0.007
Mo	1.224±0.035	1.039±0.029	1.194±0.037	1.26±0.015	1.074±0.019
φPo	0.829±0.003	0.839±0.003	0.835±0.003	0.826±0.002	0.836±0.003
φEo	0.487±0.006	0.521±0.005	0.487±0.005	0.488±0.004	0.515±0.004
φRo	0.214±0.003	0.215±0.006	0.22±0.006	0.21±0.001	0.208±0.006
Ψ_{Eo}	0.587±0.006	0.621±0.006	0.584±0.005	0.59±0.004	0.617±0.005
RC/CS	0.312±0.01	0.361±0.008	0.33±0.011	0.291±0.005	0.364±0.005
ABS/RC	3.573±0.078	3.264±0.072	3.43±0.085	3.721±0.04	3.356±0.058
TRo/RC	2.960±0.055	2.737±0.054	2.863±0.062	3.075±0.03	2.803±0.041
ETo/RC	1.736±0.03	1.698±0.032	1.67±0.03	1.815±0.023	1.729±0.031
DIo/RC	0.612±0.023	0.527±0.019	0.567±0.023	0.646±0.013	0.553±0.019
REo/RC	0.764±0.018	0.701±0.013	0.754±0.021	0.781±0.01	0.695±0.016
PIabs	1.955±0.116	2.642±0.135	2.098±0.114	1.850±0.051	2.453±0.082

Notes: These are the absolute values corresponding to the relative values shown in Figure 2A. Data are presented as means ± SE (n = 3).
